# Proteomic Differences between *Listeria monocytogenes* Isolates from Food and Clinical Environments

**DOI:** 10.3390/pathogens3040920

**Published:** 2014-12-12

**Authors:** Ge Huang, Susan L. Mason, J. Andrew Hudson, Stefan Clerens, Jeffrey E. Plowman, Malik A. Hussain

**Affiliations:** 1 Department of Wine, Food and Molecular Biosciences, Lincoln University, Lincoln 7674, New Zealand; E-Mails: annhuage@hotmail.com (G.H.); sue.mason@lincoln.ac.nz (S.L.M.); 2 Food Programme, Institute of Environmental Science Research, Christchurch 8540, New Zealand; E-Mail: ahudson999@gmail.com; 3 AgResearch Ltd., Lincoln 7608, New Zealand; E-Mails: Stefan.Clerens@agresearch.co.nz (S.C.); Jeff.Plowman@agresearch.co.nz (J.E.P.)

**Keywords:** proteomics, *L. monocytogenes*, foodborne pathogen, stress proteins

## Abstract

*Listeria monocytogenes* is an organism associated with a wide range of foods. It causes listeriosis, a severe illness that mainly affects people with weakened immune systems. Proteomic profiles of three different *L. monocytogenes* isolates were studied using 1D SDS PAGE, 2DE and mass spectrometry. The protein banding patterns generated by 1D SDS PAGE of three strains of *L. monocytogenes* were found to be similar. Visual observations from 2DE gel maps revealed that certain spots appeared to have intensity differences. Key differences in proteins synthesis of three strains of *L. monocytogenes* were found using the PDQest TM 2DE Analysis software. Comparison showed that the clinical isolate (strain SB92/844) had 53.4% and 53.9% protein profile similarity with dairy isolate (strain V7) and seafood isolate (SB92/870), respectively. The identity of selected protein spots was achieved using MALDI-TOF and ion trap mass spectrometry. It was found that certain identified proteins (*i.e.*, a major cold shock protein and superoxide dismutase) were expressed differently between two local strains of *L. monocytogenes* (SB92/844, SB92/870) and one strain from overseas (V7).

## 1. Introduction

The genus, *Listeria*, is classified in the family Listeriaceae (previously known as *Corynebacteriaceae*) with microbiological features, such as non-spore forming, catalase-positive and oxidase-negative. *Listeria* comprises a genus of Gram-positive bacteria with low GC-content, which are closely related to the genera, *Bacillus*, *Clostridium*, *Enterococcus*, *Streptococcus* and *Staphylococcus* [1,2]. The five species belonging to the genus, *Listeria*, are *L. monocytogenes*, *L. ivanovii*, *L. seeligeri*, *L. welshimeri* and the recently found species, *L. grayi* [3,4].

*L. monocytogenes* is the causative agent of listeriosis, which is one of the most serious foodborne illnesses worldwide. It was recognized as a foodborne pathogen following the listeriosis outbreak in the maritime provinces of Canada in 1983 [5]. Twenty to thirty percent of clinical infections of listeriosis result in death [1,6]. This fatality rate from listeriosis is greater than the cases of *Clostridium botulinum*. *L. monocytogenes* is distributed widely in the environment. Raw, cooked and processed foods can be a mode for the transmission of infection. *L. monocytogenes* has the ability to survive very well under freezing temperatures and other adverse growth conditions, e.g., high salt concentration, a wide range of pH and temperatures, low water activity and can form biofilms. The most dangerous feature of *L. monocytogenes* is that it can grow at low temperatures. Refrigeration reduces or prevents the growth of most food-poisoning bacteria; but, this is not true in the case of *L. monocytogenes* [7]. Moreover, *L. monocytogenes* has shown resistance to a number of sanitizers, including ethanol, sodium hypochlorite, sodium hypochlorite with methanol and quaternary ammonium compounds. These chemical agents are ineffective at reducing the numbers of *L. monocytogenes*. The ability of *L. monocytogenes* to withstand, adapt, survive and grow under stressful conditions is a major contributing factor toward the seriousness of listeriosis.

Several studies have attempted to use proteomic techniques to detect foodborne pathogens, especially *L. monocytogenes* [2,8,9,10,11,12,13,14]. Two-dimensional (2DE) polyacrylamide gel electrophoresis is the most commonly used technique to study protein changes in *L. monocytogenes* in response to stresses, such as resistance to antimicrobial chemicals, low pH, high salinity or cold shock. With the development of quantitative proteomics, high-throughput gel- and non-gel-based protein fractionation techniques coupled with protein identification by high-throughput tandem mass spectrometry (MS/MS)-based automated software algorithms are now widely used to analyze protein expression in *L. monocytogenes.* For example, Hefford* et al.* [15] identified higher levels of protein expression in biofilms of *L. monocytogenes* using 2DE analysis combined with the matrix-assisted laser desorption ionization-time of flight (MALDI-TOF) MS and MS/MS.

The development of intracellular and extracellular proteome maps of *L. monocytogenes* has been attempted using multiplexing fluorescent two-dimensional fluorescence difference gel electrophoresis (2D-DIGE) and MALDI-TOF [16,17]. More recent investigations used advanced proteome analysis techniques, such as two-dimensional nanoliquid chromatography coupled to ion-trap mass spectrometry (2DnLC-MS/MS) and multidimensional protein identification technology (MuDPIT), to study *L. monocytogenes* protein expression [18,19,20]. Most of these studies focused primarily on the identification of *L. monocytogenes*, analysis of the cell wall and secretory proteins, development of a partial proteome reference map, bacterial virulence or bacterial protein expression under strict growth condition.

There are limited numbers of proteomics studies that attempt to explore the unique protein machinery of *L. monocytogenes* strains isolated from different ecological niches [21]. This study uses comparative proteomic analysis to identify key differences in proteomic profiles of the three *L. monocytogenes* strains; two New Zealand isolates (one from seafood and another from a patient’s blood) and one overseas strain (USA) isolated from milk. To our knowledge, there is no published research on proteomic analysis of *L. monocytogenes* isolates from New Zealand. Current research on the proteomics of *L. monocytogenes* provides a promising future to develop detection technologies based on specific protein markers.

## 2. Results and Discussion

### 2.1. Growth and 1D SDS PAGE Profiles

**Figure 1:**
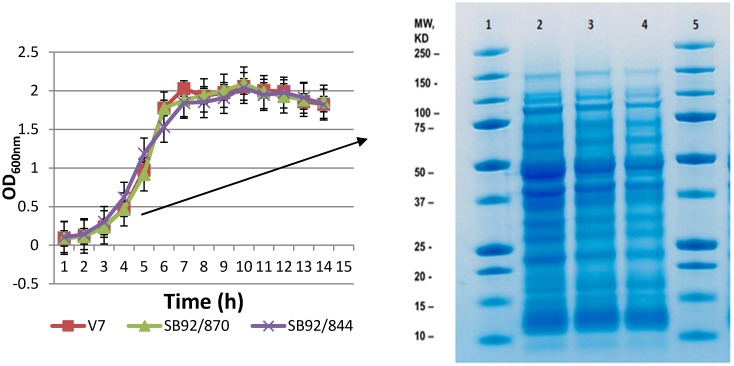
Growth of three strains of *L. monocytogenes* (V7, SB92/870, SB92/844) in brain heart infusion (BHI) medium at 37 °C (**A**) until the bacterial growth reached the stationary phase; (**B**) 1D SDS-PAGE gel of the three strains of *L. monocytogenes* showing the banding pattern of each strain. Lanes 1 and 5, protein marker; Lane 2, strain SB92/844; Lane 3, strain SB92/870; Lane 4, strain V7.

In this study, three strains of *L. monocytogenes*, SB92/844, SB92/870 and V7, were studied. The strain SB92/870 was isolated from New Zealand local seafood products (mussels) and the other strain, SB92/844, was a clinical isolate. The V7 strain was isolated from USA milk products and has been extensively studied. Firstly, the growth of the bacteria was monitored using three biological replicates for each bacterial strain. All three strains showed identical growth patterns when growth conditions were kept similar (Figure 1A). Under this growth condition, the exponential increase in OD_600_ for all three strains occurred between 2 and 7 h of incubation at 37 °C. The strains SB92/870 and V7 tended to grow a little slower than the strain SB92/844 during the exponential phase. Interestingly, strains SB92/870 and V7 achieved the same OD_600_ value of 1.77, and this was slightly higher than SB92/844 (1.53). The strain V7 first reached its highest OD (average OD_600 nm _= 2.03) in 7 h, whereas the average OD of the other two strains was about 1.9. After 7 h of growth, the strain V7 reached the stationary phase, where the OD_600_ values fluctuated between 1.83~2.7. Strains SB92/844 and SB92/870 still had very slow growth after 8 h, and the highest OD_600_ values (2.09 and 2.04, respectively) were observed in 9 h (Figure 1A). After 9 h of growth, strains SB92/870 and SB92/844 tended to reach the stationary phase with OD_600_ values from 1.9 to 2.03. In general, all three strains of bacteria had a similar growth trend during the lag and exponential phases.

For the purpose of proteomic analysis, protein samples from bacterial cells harvested at the mid-exponential phase (OD_600 _~ 1.0) were separated by first-dimension sodium dodecyl sulfate polyacrylamide gel electrophoresis (1D SDS PAGE) and visualized by Coomassie Brilliant Blue G250. No visual differences were observed in the banding patterns of the three strains on 1D gel (Figure 1B). This similarity in the banding pattern demonstrated that all strains belong to one species.

### 2.2. 2DE Proteome Profiles

**Figure 2:**
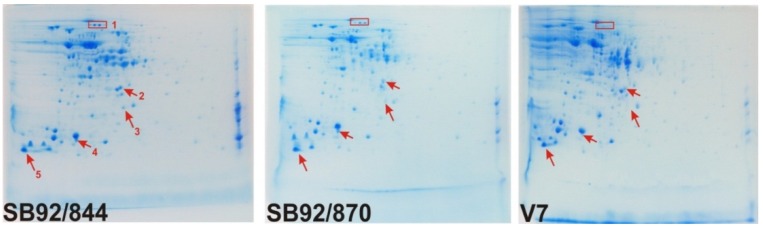
2DE gels maps of three strains of *Listeria monocytogenes* to indicate some of the differences in protein spots observed visually. (e.g., Spot Group 1, Spot 2, Spot 3, Spot 4 and Spot 5).

As the initial investigation into the protein composition of three strains of *L. monocytogenes* using 1D SDS PAGE showed no major differences, the study was extended to analysis by 2DE. This was carried out by running gels of three biological replicates for each strain, resulting in a total of nine 2DE gel maps of the three strains. Comparison of the 2DE gels showed differences between the strains of *L. monocytogenes* based either on positional shifts within the gel map or changes in intensity (Figure 2).

From comparative spot analysis, performed with PDQest, a total of 189 spots were detected on the reference gel (SB92/844). A comparison of the reference strain, SB92/844, with V7 showed that there were 101 spots that matched and 88 spots that were absent in strain V7. Comparison between the strains SB92/844 and SB92/870 revealed that 102 spots matched, whereas 87 spots were absent in the strain SB92/870.

**Figure 3:**
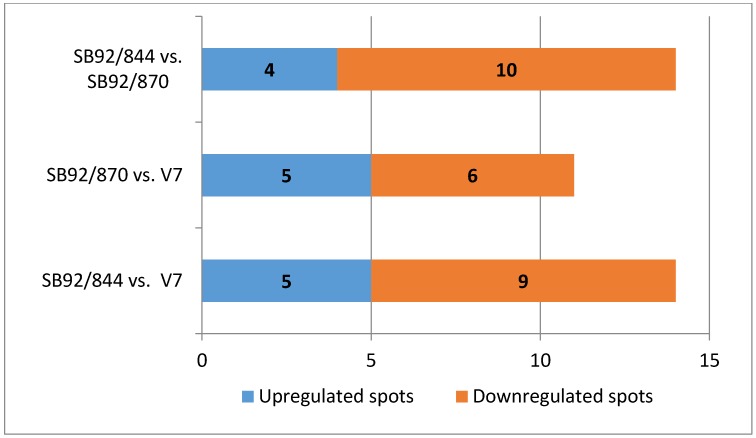
Graphical presentation of the number of differentially expressed protein spots between the strains. Data acquired through spot analysis using PDQuest (v 7.0.0) software.

Based on a relative protein peak ratio of 1.5-fold (for upregulated proteins) or 0.667 (for downregulated proteins), five protein spots were found to have increased in intensity in the strain SB92/844 relative to V7, one spot in particular of a major cold-shock protein (Table 1) with a high ratio of +3.5. The levels of nine proteins spots decreased in SB92/844 (Figure 3); the one showing the most decrease being that of 4203 with a ratio of −3.1 (data not shown). The relative protein expression data revealed five protein spots that were clearly more prevalent in strain SB92/870 in comparison to V7, while six protein spots were less prevalent in SB92/870 relative to V7. When both New Zealand isolates were compared, four protein spots were more prevalent in the clinical isolate SB92/844 and 10 less prevalent than in SB92/870.

**Table 1:** Comparative expression of identified proteins in *Listeria monocytogenes* strains.

Spot No. ^a^	Identified Protein	SB92/844 *vs.* V7 ^b^	SB92/870 *vs.* V7 ^c^	SB92/844 *vs.* SB92/870 ^d^
0001	Major cold-shock protein	Protein only present in SB92/844		
0002	Major cold-shock protein, partial	↑(+1.8)	↑(+1.7)	(+1.0)
0004	Major cold-shock protein, partial	(+1.2)	↑(+1.7)	(−1.3)
1001	Major cold-shock protein, partial	(−1.1)	(+1.4)	↓(−1.6)
1003	50S ribosomal protein L7/L12	↑(+2.0)	(+1.2)	↑(+1.6)
1103	Regulatory protein SpoVG	(+1.0)	(+1.3)	(−1.3)
2001	co-chaperonin GroES	↓(−1.9)	↓(−1.8)	(−1.1)
2402	CD4+ T-cell-stimulating antigen	Protein not present in V7, but present in SB92/870		↓(−1.7)
3004	Phosphocarrier protein HPr	(+1.2)	↑(+1.5)	(−1.3)
3005	Phosphocarrier protein HPr	Protein not present in V7, but present in SB92/870		↓(−1.7)
5802	Glyceraldehyde-3-phosphate dehydrogenase	Protein not present in V7, but present in SB92/870		(−1.1)
5803	Glyceraldehyde-3-phosphate dehydrogenase	Protein not present in V7, but present in SB92/870		(+1.1)
6102	Superoxide dismutase	↓(−1.6)	↓(−2.0)	(+1.3)
6205	Ribosome recycling factor	↑(+2.3)	↑(+1.7)	(+1.3)
6502	Glyceraldehyde-3-phosphate dehydrogenase	↓(−3.0)	↓(−3.1)	(+1.1)
6603	Glyceraldehyde-3-phosphate dehydrogenase	(+1.0)	↓(−2.6)	↑(+2.6)
7102	PTS system, glucose-specific, IIA component, putative	Protein not present in V7, but present in SB92/870		↓(−4.2)
7201	Phosphoglycerate mutase	↓(−1.9)	Protein not present in SB92/870	
9203	Major cold-shock protein	↑(+3.5)	↑(+1.5)	↓(−5.4)

^a^ Spot number marked on the reference gel. ^b^ Relative protein expression values in the strain SB92/844 compared to the strain V7. ^c^ Relative protein expression values in the strain SB92/870 compared to the strain V7. ^d^ Relative protein expression values in the strain SB92/844 compared to the strain SB92/870.

In a previous proteomic study of *L. monocytogenes* utilizing 2DE, hundreds of protein spots were observed in a single gel [22]. In this study, 2DE mapping of the protein profiles of the three strains of *L. monocytogenes* cultivated under the same growth condition revealed that the majority of the protein spots were localized in a region between pH 4 and pH 6. In comparative proteomics analysis of the *L. monocytogenes* strain V7 to strain SB92/844, 52.4% of detected spots were present only in strain V7 and 54.0% unique spots were found in *L. monocytogenes* strain SB92/870. An early study conducted in 1995 demonstrated that 46.7% of the spots were found to be common between *L. monocytogenes* strains across serotypes, and serotypes 1/2a and 1/2b were in two different major clusters [23].

### 2.3. Identification of Proteins by MALD-TOF MS and Ion Trap MS

**Figure 4:**
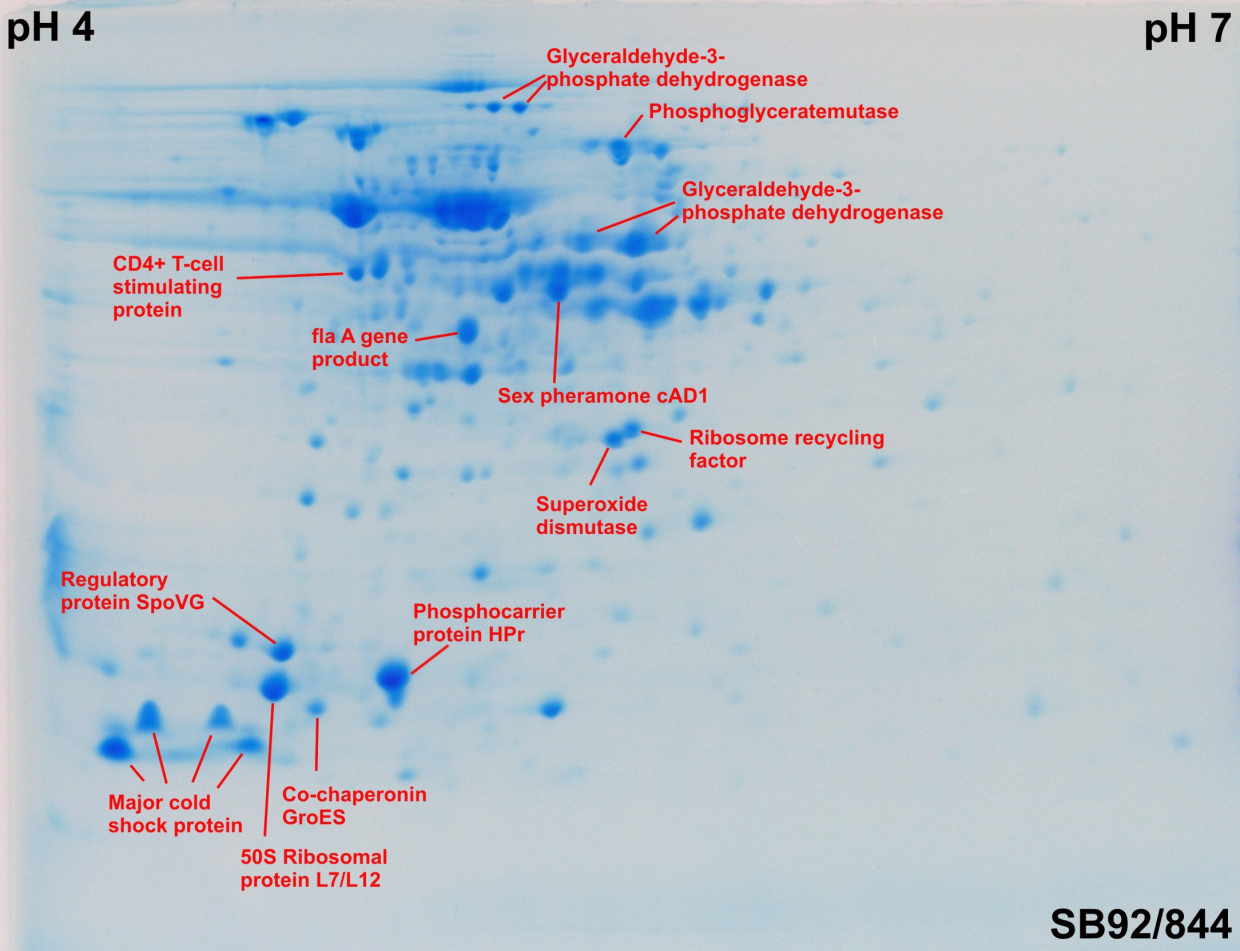
A 2DE map of *L. monocytogenes* strain SB92/844.

Mass spectrometry analysis using MALD-TOF and ion trap MS and subsequent matching with ProteinScape resulted in the identification of proteins in 19 of the excised spots (Figure 4). Of these, four were cold shock proteins that play a role in detoxification and adaptation to atypical conditions. Three of the protein spots were co-chaperonin GroES, phosphocarrier protein HPr and CD4+ T-cell-stimulating antigen, which function as transport/binding proteins, lipoproteins and membrane bioenergetics, respectively. Five of the identified protein spots were members of the carbohydrate metabolism group, specifically phosphoglycerate mutase, oligopeptide-binding protein and glyceraldehyde-3-phosphate dehydrogenase. The remaining identified spots were involved in protein synthesis, protein folding, oxidative responses and steroid biosynthetic processes.

Of the identified proteins (Figure 4), Spot Group 1 (Figure 2) contains two proteins, oligopeptide ABC transporter and oligopeptide-binding protein, which are absent in strain V7. Spot 2 was identified as a ribosome recycling factor, and this was found to undergo a positional shift in the strain SB92/870. Spot 5, which matched a major cold-shock protein that has a role both in detoxification and adaptation to atypical conditions, has undergone a positional shift in the strain SB92/844.

### 2.4. Comparative Analysis of Up and Down Regulated Proteins

Of the two *L. monocytogenes* isolates from New Zealand, strains SB92/844 and SB92/870, and the USA strain, V7, 19 proteins were identified (Table 1). In the case of the New Zealand isolates, five were synthesized in high amounts in SB92/844 relative to V7, of which three were the major cold-shock protein, 50S ribosomal protein L7/L12 and the ribosome recycling factor. A further four proteins showed a decrease in level relative to V7, and these were co-chaperonin GroES, superoxide dismutase (SOD), glyceraldehyde-3-phosphate dehydrogenase and phosphoglycerate mutase. Another four proteins were not differentially expressed in SB92/844 relative to V7, specifically two major cold shock proteins, regulatory protein SpoVG and glyceraldehyde-3-phosphate dehydrogenase. Of those proteins, spots identified in strain SB92/870 (Table 1), three out of 19 identified proteins had lower expression, specifically two spots assigned to glyceraldehyde-3-phosphate dehydrogenase and one to a major cold shock protein. Three major cold-shock proteins showed upregulation in SB92/870. One of the major cold-shock proteins was not present in strains SB92/844 and V7 (Spot 0001). Among the identified proteins, five were not present in V7 strain, but two were present in the strains SB92/844 and SB92/870. These proteins are CD4+ T-cell-stimulating antigen, phosphocarrier protein HPr, glyceraldenhyde-3-phosphate dehydrogenase and glucose-specific IIA component.

Of those proteins identified, glyceraldehyde-3-phosphate dehydrogenase and phosphoglycerate mutase are responsible for core metabolic functions under the same growth condition. Phosphoglycerate mutase is involved in catalyzing the isomerization of phosphoglycerate substrates, a process essential for the metabolism of glucose and/or 2,3-phosphoglycerate [24,25]. Previous studies have indicated that strains from clinical cases are more virulent than those found in food [26]. Thus, though the two local strains have a similar proteomic profile, they differ in the relative amounts of some proteins, and this may have some impact on their virulence. Hence, differences in the expression stress response between these two local strains may have an impact on the type of environment best suited to the bacteria or how each strain is affected by the host’s immune response.

The expression of stress response proteins is very important for the survival of *L. monocytogenes* under stress, such as cold, heat, osmotic, acid, alkaline and high hydrostatic pressure stress. These stress proteins also contribute to the virulence and pathogenicity. For example, one study has shown, from comparisons between the cold stress wild-type and mutants lacking *csp* genes in the *L. monocytogenes* EDGe strain, that *csp* genes play an important role in cell invasion at temperatures of 37 °C [27]. The virulent genes and cell invasion of *L. monocytogenes* are controlled by the expression of prfA, as shown by the decrease in expression of this gene during long-term cold storage at 4 °C, which has demonstrated that this is temperature dependent and associated with pathogenicity [28,29,30]. The above examples suggest that temperature-dependent virulence gene expression/repression, as well as membrane damage and cell surface modifications in these organisms exposed at low temperatures might lead to phenotypic virulence defects observed in cold-adapted *L. monocytogenes*. Comparison of the proteomes between *L. monocytogenes* cells grown in human THP-1 monocytes and in trypticase soy broth (TSB) broth using 2D-DIGE indicated that the general stress protein, Ctc, and oxidative stress protein, SOD, play an important role in survival and adaptations for intracellular uptake [17]. Several major cold-shock proteins were differentially expressed between two local strains during the log phase. This may indicate that these cold-shock proteins may contribute to the virulence and pathogenicity of *L. monocytogenes* strain SB92/844. The cold shock proteins (Csp) function as nucleic acid chaperones, which can bind RNA and DNA. They play important roles in the regulation of various microbial physiological processes, such as replication, transcription and translation in the bacterial cells [31,32].

Additionally, although those identified proteins were expressed in both local strains, the expression of many of them was significantly higher in SB92/870 than in SB92/844. Such proteins may play an important role for the bacteria to survive under stress conditions.

## 3. Experimental Section

### 3.1. Bacterial Strains and Growth Conditions

Three *L. monocytogenes* strains (New Zealand isolates, SB92/870 and SB92/844, and strain V7) have been used in this study. Strain SB92/870 was isolated from smoked mussels, while SB92/844 is a clinical isolate from a patient’s blood. The V7 strain was isolated from USA milk products, and has been extensively studied [33]. The bacterial strains were purchased from the Institute of Environmental Science and Research Ltd (ESR) of New Zealand.

Bacteria were streaked out onto brain heart infusion (BHI) agar, (Oxoid, Basingstoke, UK) at 37 °C for 24 h. Colonies were picked to inoculate into BHI broth (Oxoid) and incubated at 37 °C overnight [34]. Bacterial overnight cultures were transferred into 250 mL of BHI broth with an initial OD_600_ ~ 0.1 and grown at 37 °C under aerobic conditions using rotary aeration (130 rpm). Bacterial growth was monitored by measuring OD_600_ every hour, until it reached the stationary phase (~13 h). For proteomic analysis, cells were collected at the exponential phase (OD_600_ ~ 1.0). The bacterial cultures were pelleted by centrifugation at 5,600× *g* for 10 min at 4 °C. Cell pellets were washed with PBS twice.

### 3.2. Protein Extraction

The cell pellets were suspended in 4 mL of lysis solution (2% Triton X-100, 2.6 mg/mL sodium azide, 0.1M Tris (pH 8.0), 8 mM phenylmethanesulfonyl fluoride) and then incubated on ice for 1.5 h. Samples were treated with 2 μL (10,000 U/mL) DNase I and 2 μL of 20 mg/mL RNase A for 30 min at 37 °C, and 0.5 mL of solubilization buffer (7 M urea, 20 mM Tris-Cl, pH 8.0, 5 mM EDTA, 5 mM MgCl2, 4%-CHAPS, 1 mM phenylmethanesulfonyl fluoride) was added. Cell debris was removed by centrifugation (18,000× *g* for 5 min at 4 °C). Protein-rich supernatants were stored at −80 °C until further use. The protein concentration was determined using the BCA kit according to the manufacturer’s protocol.

### 3.3. 1D SDS PAGE

For 1D SDS PAGE analysis, 30 μg of the protein from each strain were loaded onto a Criterion gel (Bio-Rad, Hercules, CA, USA) along with 5 μL of Precision Plus Protein Standards (Bio-Rad). The samples were run at 200 V, 80 mA and 15 W for 50 min and visualized using colloidal Coomassie Brilliant Blue G250 (Bio-Rad).

### 3.4. 2DE and MS Analysis

2DE of the protein extracts from the three *L. monocytogenes* strains was performed according to the protocol described by Cacace* et al.* [35], each strain being run in triplicate. About ~810 μg of each protein extract was separated in the first dimension in a Protean IEF (Bio-Rad Laboratories, Hercules, CA, USA) on linear 18-cm pH 4–7 immobilized pH gradient strips. The proteins were separated in the second dimension on 2 mm × 18 cm × 20 cm 15% T Trix-glycine polyacrylamide gels in a Protean IIxi tank (Bio-Rad Laboratories).

Protein spots were visualized by staining with colloidal Coomassie Brilliant Blue-G250. Spot detection, quantization and analysis were performed using the PDQuestTM 2D Analysis software, version 7.0.0 (Bio-Rad).

Spots were excised from the gels and destained by washing twice with a solution of 200 mM ammonium bicarbonate and 50% acetonitrile (AcN) for 1 h at 37 °C. The spots were reduced with a solution of 50 mM Tris (2-carboxyethyl) phosphine and 100 mM ammonium bicarbonate at 56 °C for 45 min. Following this, they were shaken with 150 mM iodoacetamide in 100 mM ammonium bicarbonate for 30 min. They were then shaken with AcN for 10 min and dried on a Centrivap vacuum centrifugal concentrator (Labconco, Kansas City, MI, USA). After digestion with 2 μg tosyl phenylalanyl chloromethyl ketone-trypsin in 50 mM ammonium bicarbonate:AcN (8.625:1.375) for 18 h at 37 °C, peptides were extracted from the gel by vortexing for 3 h in the presence of Empore^TM^ disks that had been pre-wetted with AcN and methanol, following which the peptides were extracted with 75% AcN in 0.1 mM trifluoroacetic acid (TFA). The extracts were dried down on a Centrivap vacuum centrifugal concentrator and reconstituted in 0.1% TFA.

Samples were prepared for MALDI-TOF by applying 1 mL of a α-cyano-4-hydroxycinnamic acid (CHCA)-saturated solution of 97:3 acetone:0.1% TFA to an AnchorChip plate and immediately removed. The sample was applied as 1 mL dissolved in 0.1% TFA, allowed to incubate for 3 min before removal, followed by the addition and immediate removal of 2 mL of 0.1% TFA. Finally, 1 mL of a solution of 0.1 mg/mL CHCA in 6:3:1 ethanol:acetone:0.1% TFA was added and allowed to dry. MS and MS/MS spectra were collected on an ultraflex III MALDI-TOF/TOF mass spectrometer, (Bruker Daltonik, Bremen, Germany). Spectra were calibrated externally using a Peptide Calibration Standard (Bruker) containing angiotensin II (*m*/*z* 1,046.5418) and I (*m*/*z* 1296.6848), substance P (*m*/*z* 1,347.7354), bombesin (*m*/*z* 1,619.8223), ACTH-clip (*m*/*z* 2,093.0862), ACTH-clip (*m*/*z* 2,465.1983) and somatostatin (*m*/*z* 3,147.471) diluted six-fold with matrix solution.

LC-MS/MS was performed on a nanoAdvance UPLC coupled to an amaZon speed ETD mass spectrometer (Bruker Daltonik) equipped with a CaptiveSpray source (Bruker Daltonik, Bremen, Germany). Two microliters of sample were loaded on a C18AQ nano trap (Bruker, 75 μm × 2 cm, C18AQ, 3-μm particles, 200-Å pore size). The trap column was then switched in line with the analytical column (Bruker Magic C18AQ, 100 μm × 15 cm C18AQ, 3-μm particles, 200-Å pore size). The column oven temperature was 50 °C. Elution was with a gradient from 0% to 40% B in 90 min at a flow rate of 800 nL/min. Solvent A was LCMS-grade water with 0.1% formic acid and 1% AcN; Solvent B was LCMS-grade AcN with 0.1% FA and 1% water. Automated information dependent acquisition (IDA) was performed using Hystar PP 3.2.44.0 software, with an MS survey scan over the range *m*/*z* 50–2200, followed by three MS/MS spectra from 50–2,200 *m*/*z* acquired during each cycle of a 30-ms duration.

### 3.5. Protein Identification and Classification

After the MALDI-TOF MS and MS/MS data acquisition, peak lists were extracted from the data using FlexAnalysis software (Bruker). These peak lists were searched against the NCBInr database using ProteinsScape 3.1.0 (Bruker) with an in-house MASCOT server (Matrix Science, London, U.K.). The following search parameters were used: semitrypsin was chosen as the proteolytic enzyme; standard modifications were carbamidomethylation (C) and oxidation (M) peptide; fragment ion tolerances were set at 0.2 Da; taxonomy was restricted to *Firmicutes* (Gram-positive bacteria); and two missed cleavages were allowed. Following each LC-MS/MS run, peak lists were queried against *Firmicutes* in the UniProt database using the Mascot search engine (v2.2.03, Matrix Science) maintained on an in-house server. The Mascot search parameters included semitrypsin as the proteolytic enzyme with two missed cleavages; standard modifications were carbamidomethylation (C), deamidation (NQ) and oxidation (M); error tolerance was set to 0.15 Da for MS and 0.3 Da for MS/MS. Search results were compiled and analyzed using ProteinScape 3.1.0 (Bruker) using the ProteinExtractor function and automatic assessment of true and false positive identifications of protein and peptide matches. Acceptance thresholds for peptide and protein scores were set at 25 and 80, respectively. Results assessed as being true matches were used for further analysis. Error-tolerant searches were run on all of the spots in the Type I keratin region, all parameters being kept the same, except the enzyme was set to trypsin and no modifications were searched for.

The functionalities of identified proteins were searched for using online bioinformatics resources.

## 4. Conclusions

This study presents an initial view of the protein expression in two strains of *L. monocytogenes* species in New Zealand. This study found that two local strains, SB92/844 and SB92/870, had identical growth profiles in comparison to the overseas strain under the same growth conditions. From 1D SDS PAGE, it was apparent that the protein banding patterns of three strains of *L. monocytogenes* were very similar with no significant differences in the intensities in bands in the gel. In contrast, 2DE gels maps and comparative spot analysis revealed that strains exhibited differences in the relative levels of some proteins when their respective strains were grown under similar physiological conditions. Furthermore, this study also highlighted the possibility that the stress response proteins of *L. monocytogenes* may have a role in the clinical behavior and pathogenesis of specific strains. This study points to the need for further studies to be carried out to verify whether differential expression of cold shock proteins may be associated with the virulence and pathogenicity of *L. monocytogenes* under various growth conditions, such as different media, temperatures, pH and stress responses.
